# Supplementary material for: Revealing biases in the sampling of ecological interaction networks
Source: PeerJ. 2019 Sep 2;7:e7566. doi: 10.7717/peerj.7566 (PMC6727833; doi:10.7717/peerj.7566)

Appendix S1: Generating networks with *NetGen*

This section describes the different types of networks and modules constructed by the script *NetGen*, available in Python and, and within the package *EcoNetGen* in R (https://doi.org/10.5281/zenodo.1212559), now available on CRAN (https://CRAN.R-project.org/package=EcoNetGen). Basic input parameters are network size (*N*) here defined as total number of nodes, average module size (M_{av}), average degree (*k*), and type of modules. *NetGen* creates a network with these properties, which are specified by the user, along with the types of modules the network will contain. There are six basic types of modules that can be specified: random, scale-free, nested, bipartite-nested, bipartite random, tri-trophic nested and tri-trophic random. Networks may also contain a mixture of module types. Once the modules have been created, the nodes within each module can be randomly rewired with probability p_{local}. Moreover, the nodes of the full network can be further rewired with probability p_{rew} in order to create connections among the modules.

Module sizes (*M*) are taken from a negative exponential distribution, $\rho(M)$, with average M_{av}.

$$\rho(M) = \frac{1}{M_{av}} e^{-M/M_{av}}$$

eq. A1

If M_{av}=*N*, the program generates a network with single module. In order to avoid the construction of modules that are too small to usefully model ecological networks, we also introduced two control parameters that can be specified by the user, *ModCutOff* and

SubModCutOff, that are the smallest acceptable values for a module or submodule (in the case of bipartite or tri-trophic modules) respectively.

Module Types

Module types can be uniform (all modules of the same type; all scale-free, for instance) or mixed. In the latter case, each module type is chosen with probabilities specified by the user, so that only modules of certain types can be generated if the probability of the others is set to zero. Each module type and the methods used to construct it are described below. In all cases a binary, symmetric adjacency matrix is generated with zeros and ones as a representation of the resulting network structure.

1. Random: Given the module size M and the average degree k, we define the probability:

$$\rho = \frac{k}{M-1} \qquad \text{eq. A2}$$

such that any two nodes in the module are connected with probability ρ.

2. Scale-free

We use the Barabási-Albert preferential attachment procedure (Barabási and Albert, 1999) starting with a fully connected network with m_0 nodes, extra nodes are added one by one until the module size is M. Each new node added to the network has m connections with the already existing nodes and the probability p_i that an existing node i receives a connection is proportional to its degree:

$$p_i = \frac{k_i}{\sum_j k_j} \qquad \text{eq. A3}$$

The program sets $m=k/2$ and $m_0 = k+1$.

3. Nested

In nested networks, there is a hierarchy in the way nodes interact with each other. In a perfectly nested network, node 1 interacts with nodes 2,3, ...,r_1; node 2 interacts with nodes 1, 3, 4, ... r_2; and so on, where $r_1 > r_2 > r_3$ .... The higher the label of the node, the fewer interactions it will have. In order to reconcile this structure with the given average degree k we need to specify how the number of connections r_n of a node depends on its label n. Here we chose a negative exponential dependency

$$r_n = M e^{-\alpha(n-1)} \qquad \text{eq. A4}$$

where α is a parameter that will be adjusted according to the average network degree. With this definition, the node labeled n=1 connects with the other nodes and has M-1 connections; node n=2 has $M\, exp(-\alpha)$ connections and so on. The total number of connections between the nodes is:

$$\sum_{n=1}^{M}\left[M e^{-\alpha(n-1)} - 1\right] = M\left(\frac{1-e^{-M\alpha}}{1-e^{-\alpha}} - 1\right) \qquad \text{eq. A5}$$

where the last term (-1) refers to the fact that a node does not interact with itself. The average number of connections is, therefore

$$k = 2\left(\frac{1-e^{-M\alpha}}{1-e^{-\alpha}} - 1\right) \qquad \text{eq. A6}$$

Assuming $M\alpha >> 1$, we can neglect the exponential in the numerator and obtain a closed expression for the parameter α:

$$\alpha = \log\left(1 + \frac{2}{k}\right) \qquad \text{eq. A7}$$

which is the value used by the program. Note that if k is not too small we can further approximate $\alpha \sim 2/k$ and the condition $M\alpha >> 1$ becomes $M >> k/2$, *i.e.*, the module size has to be larger than the average degree of the network. Therefore, α scales with the inverse of the average number of interactions: groups of nested species with large number of interactions are modeled by small α and vice-versa. The condition $M >> k/2$ is likely to hold for nested modules.

4. Bipartite Nested

This type of structure describes the interaction between two groups of species (like plants and insects) where individuals of one group only interact with individuals of the other group for a specified type of interaction (like pollination). Here, the module is first split into two submodules of sizes M_1 and M_2, chosen from an exponential distribution. If one of the modules is smaller than *SubModCutOff*, a new draw of sizes is performed. The two submodules define four blocks of the adjacency matrix and only the off-diagonal (inter-groups) modules are populated. We follow the nested procedure described above to populate the upper-left and lower-right blocks. The number of connections in the upper-left block nodes, $i = 1$ to M_1, is set to:

$$r_n = M_2 e^{-\alpha(n-1)} \qquad \text{eq. A8}$$

so that the total number of connections in the block is

$$M_2\left(\frac{1-e^{-M_2\alpha}}{1-e^{-\alpha}}\right)\approx\frac{M_2}{1-e^{-\alpha}} \qquad \text{eq. A9}$$

For the lower-right block we find, similarly,

$$M_1\left(\frac{1-e^{-M_1\alpha}}{1-e^{-\alpha}}\right)\approx\frac{M_1}{1-e^{-\alpha}} \qquad \text{eq. A10}$$

Adding the total number of links and dividing by the module size $M_1 + M_2$ we obtain the average degree and extract the parameter α:

$$\alpha=\log\left(1+\frac{1}{k-1}\right) \qquad \text{eq. A11}$$

To ensure that the resulting adjacency matrix is symmetric, the program constructs only the upper-left block and copies it transposed into the lower-right block.

5. Tri-Trophic Nested

The generated network is divided into three modules such that primary connections exist only between modules 1 and 2 and between modules 2 and 3. Species represented by modules 1 and 3 are only connected indirectly through species in module 2. In this case of nested species the pairs of modules 1-2 and 2-3 are *bipartite nested* pairs.

6. Tri-Trophic Random

The generated network is again divided into three modules such that primary connections exist only between modules 1 and 2, and between modules 2 and 3. In this case the pair of

modules 1-2 are connected as *bipartite nested* but the pair 2-3 are connected as *bipartite random* pairs.

Figure A1 shows examples of these constructions for small networks with 25 nodes. Nodes within each module can be randomly rewired with probability p_{local} to make them look more realistic. Nodes can be further rewired with probability p_{rew} in order to generate connections among the different modules. Figure A2 shows an example of a larger network generated by *NetGen*, in both a visual representation of its nodes and links, and its accompanying adjacency matrix.

Additional reference for Appendix S1

Barabási, A.L. and Albert, R. (1999) Emergence of scaling in random networks. *Science*, **286**, 509-512.

Figure A1. Examples of modules with 25 nodes: (a) random, (b) scale-free, (c) nested, (d) bipartite nested, (e) tri-trophic bipartite-bipartite and (f) tri-trophic bipartite-nested. Nodes can be randomly rewired with probability p_{rew} to make them look more realistic. Here we set p_{rew} = 0. Nodes are plotted by node labels, starting from the lower left corner and increasing to the right and top.

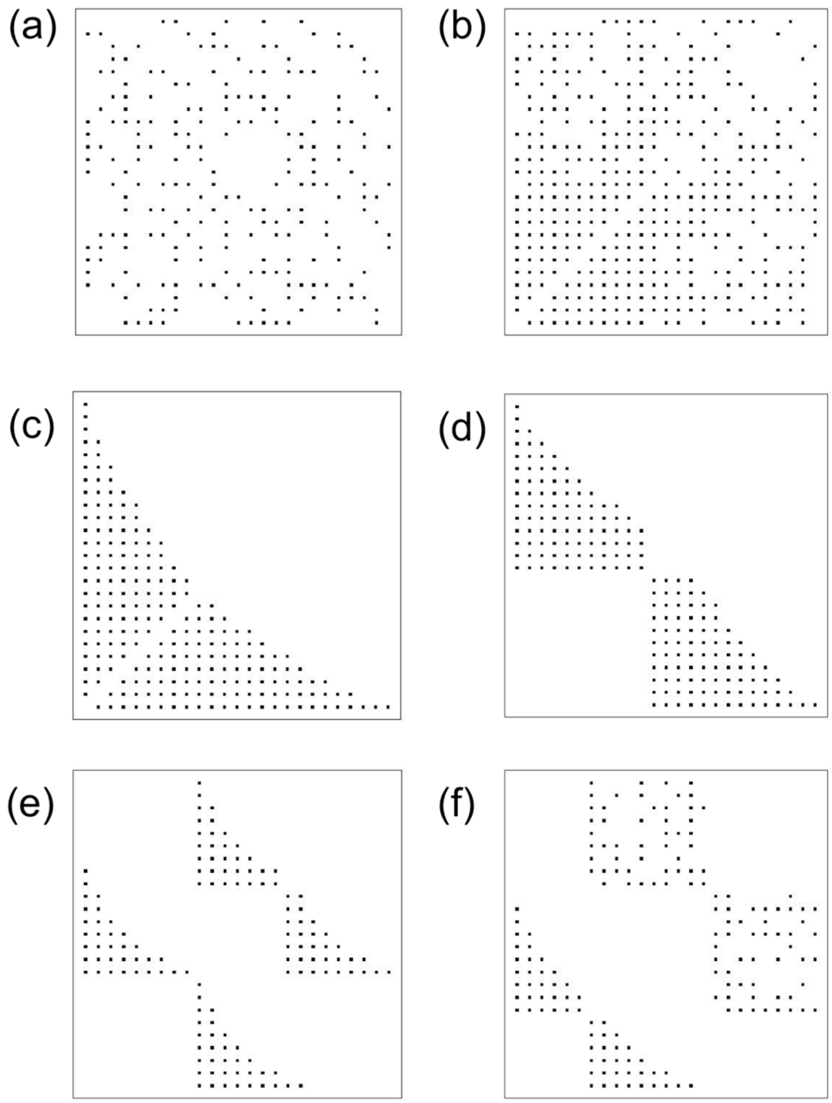

Figure A2. Generated (a) network and (b) adjacency matrix drawn with *NetGen*, using N=1000 nodes, mixed module types and average module size $M_{av}=50$.

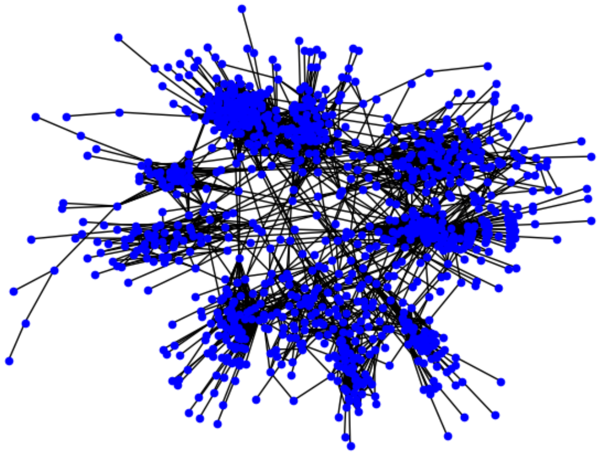

(a)

(b)

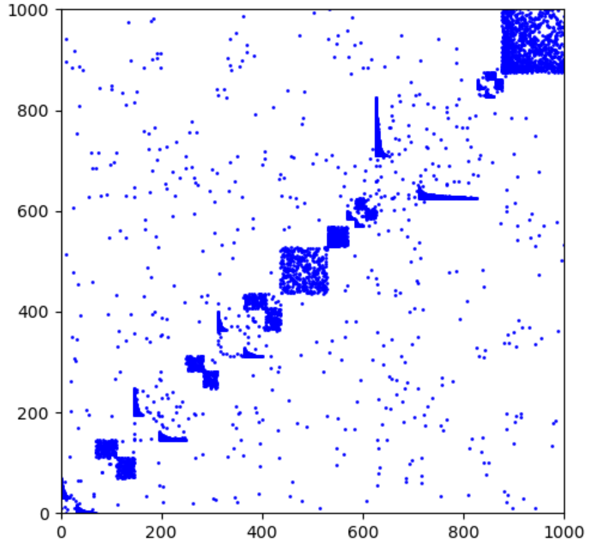

Supplement: Supplemental Information 1 [file peerj-07-7566-s001.pdf]
