# Supplementary material for: Revealing biases in the sampling of ecological interaction networks
Source: PeerJ. 2019 Sep 2;7:e7566. doi: 10.7717/peerj.7566 (PMC6727833; doi:10.7717/peerj.7566)

Appendix S2: Sampling simulated networks with *NetSampler*: an example

In this section we give a detailed example of the sampling procedure provided by the code *NetSampler*. We use the network shown in Figure A2 and sample m anchor nodes according to their degree, for m=10, 20, …, 100. Sampling by degree means that the probability of node i be selected as an anchoring node is proportional to its degree, $p(i)=k_i/k_T$, where $k_T=\sum k_i$. For each sampled anchor node, a maximum of 5 of its neighbors will be randomly picked and added to the "observed network." The observed network is the output of the sampling procedure performed on the full network. For each value of m the sampling procedure is repeated 1000 times and averages and variances are calculated. Properties of the observed network can then be analyzed and compared to those of the full network.

Figure B1 shows an example the observed network for m=100, sampling by degree. The sampled nodes are shown in red in their position in the original network and adjacency matrix. Figure B2 shows only the sampled nodes, numbered according to sample order. The first 93 nodes correspond to anchor nodes, and others nodes included are their neighbors. In the sampled network, the neighbors of anchor nodes do not connect among themselves, since only connections with the anchor nodes were sampled. This generates the two stripes in the graph of the adjacency matrix, showing that each neighbor is connected to a anchor node, and the empty block where neighbor-neighbor interactions should be.

After 1000 realizations of the procedure, a list of the average properties of the sampled networks, together with their variances, are produced (Table B1). The parameters calculated automatically are: network size (S), size of the largest connected component (LC), size of the

largest component divided by the network size (*RSLC* = *LC*/*S*), and number of connected components (*NC*).

Figure B1. Example of (a) sampled nodes within the full the network displayed in Figure A2, and (b) the full adjacency matrix showing sampled connections. The observed nodes and links are displayed in red within the full network in blue. Here, 100 anchor nodes were sampled by degree and up to 5 neighbors of each sampled anchor node were added to the network. On average (after 1000 independent samplings) the size of the observed network is 376, the size of the largest component is 221 and the number of components is 14 (table B1).

(a)

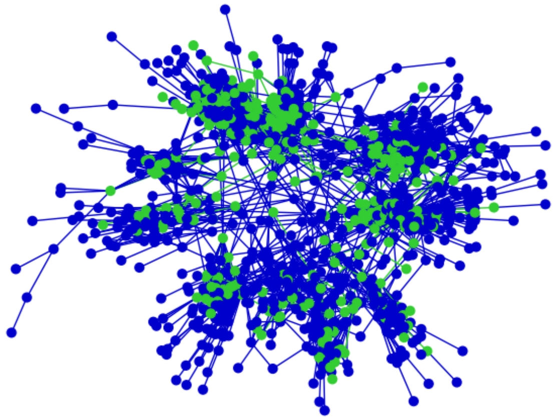

(b)

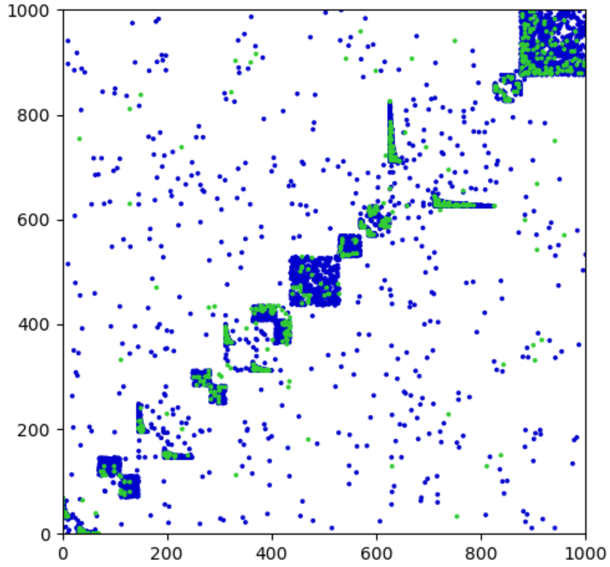

Table B1. Average values and variances of sampled network: network size (*S*), size of the largest connected component (*LC*), Relative size of the largest connected component (*RSLC*), equal to the size of the largest component divided by the network size (*RSLC* = *LC*/*S*), and number of connected components (*NC*). Values shown are for the network in Figure A2 and 1000 realizations of the sampling procedure (see text).

m	***S***	**var(*S*)**	***LC***	**var(*LC*)**	***RSLC***	**var(*RSLC*)**	***NC***	**var(*NC*)**
10	61.25	6.57	17.82	6	0.29	0.13	7.38	1.28
20	113.28	10.75	29.92	11.49	0.26	0.13	11.8	2.28
30	161.59	10.54	44.11	14.35	0.27	0.11	14.07	2.82
40	201.55	12.56	58.43	20.73	0.29	0.12	15.68	2.75
50	240.79	12.91	80.44	29.01	0.33	0.14	15.79	2.98
60	269.05	11.15	97.62	37.44	0.36	0.15	16.03	3.07
70	302.26	14.12	141.37	49.21	0.47	0.18	14.3	3.52
80	331.31	14.74	150.79	48.38	0.46	0.17	15.2	3.16
90	356.4	16.08	187.23	61.93	0.53	0.2	14.16	3.34
100	376.07	15.46	221.51	64.82	0.59	0.2	14.26	3.24

Figure B2. Plots of the (a) sampled network and (b) adjacency matrix extracted from Figure B1.

(a)

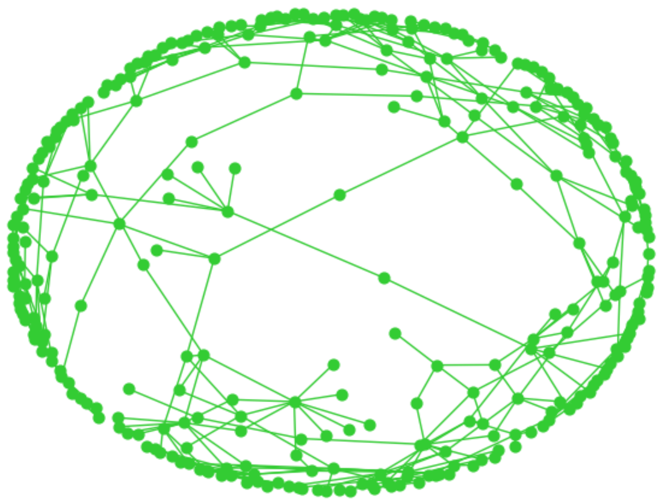

(b)

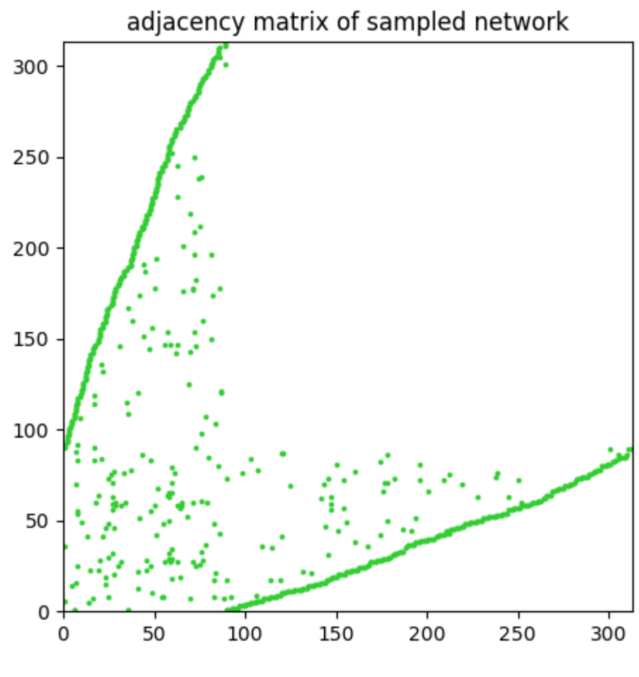

Supplement: Supplemental Information 2 [file peerj-07-7566-s002.pdf]
