# Supplementary material for: Revealing biases in the sampling of ecological interaction networks
Source: PeerJ. 2019 Sep 2;7:e7566. doi: 10.7717/peerj.7566 (PMC6727833; doi:10.7717/peerj.7566)
Supplement: Supplemental Information 3 [file peerj-07-7566-s003.pdf]

Appendix S3: The abundance distributions

In this section we show in more detail how four abundance distributions are calculated in *NetGen*. Abundances are attributed to each module separately and are optional in the script. Abundances may represent ecological information such as individuals within a species or observed number of interactions. The four abundance distributions are: negative exponential (simply referred to as "exponential"), Fisher log-series (or "Fisher"), lognormal, and a distribution based on module.

Although abundances are assigned in the generation of the networks (as a specific abundance distribution), they are only assigned if the networks are to be sampled by their abundances. In this sense, we consider the abundance distributions to be part of the sampling routine. The probability of selecting an anchor node is not deterministic, *i.e.*, the most abundant nodes are not necessarily selected first. The sampling of a node is proportional to its abundance, so that the most abundant nodes are more likely to be selected.

1. Exponential

The probability that a node has abundance x is

$$p_E(x) = \frac{1}{x_0}\exp(-x/x_0) \qquad \text{eq. C1}$$

NetSampler uses x_0=1.

2. Fisher

In this case the number of species with n individuals is $S_n = \alpha\, y^n / n$ where α is a measure of biodiversity and $0 < y < 1$ are the so called Fisher parameters. These two parameters control the shape of distribution and can be related to the total number of species

$$S = \sum_n S_n = -\alpha \ln(1-y) \qquad \text{eq. C2}$$

and total number of individuals

$$N_I = \sum_n n S_n = \alpha y / (1-y) \qquad \text{eq. C3}$$

Solving for y we obtain

$$y = \frac{N_I}{N_I + \alpha} \qquad \text{eq. C4}$$

and substituting (1-y) from eq. C4 into eq. C2 we find

$$S = \alpha \ln(1 + N_I / \alpha) \qquad \text{eq. C5}$$

Equations C4 and C5 show how α and y relate to S and N_I. The probability that a species has n individuals is, therefore, S_n/S:

$$p_F(n) = -\frac{y^n}{n \ln(1-y)} \qquad \text{eq. C6}$$

The number of species S is the size of the network, so given S and y we can calculate α (eq. C2) and N_I (eq. C5). The average number of individuals per species is

$$\langle n \rangle = \sum_n n p_F(n) = -\left(\frac{y}{1-y}\right)\frac{y^n}{n \ln(1-y)} \qquad \text{eq. C7}$$

Because sampling is performed according to relative abundances, the value of y should not matter. *NetSampler* uses $y = 0.5$.

3. Lognormal

The probability that a species has x individuals is

$$p_L(x) = \frac{1}{\sigma x\sqrt{2\pi}} \exp\left\{-\left(\log x - \log x_0\right)^2 / (2\sigma^2)\right\} \qquad \text{eq. C8}$$

where x_0 is the average number of individuals and σ is the variance. *NetSampler* uses x_0=1 and σ=0.2.

4. Module

To assign abundances by module, we first assign a different probability $P(j)$ to each module j of network. These probabilities are drawn from an exponential distribution with unit average. The probability associated to a node in module j is

$$p_M(j) = P(j) / N(j) \qquad \text{eq. C9}$$

where $N(j)$ is the number of nodes in module j. Inside each module the probabilities are constant but some modules can be much more likely to be sampled than others. The idea is to simulate the fact that some groups of species are easier to observe than others or that some researchers focus on particular types of interactions.
