# Supplementary material for: Revealing biases in the sampling of ecological interaction networks
Source: PeerJ. 2019 Sep 2;7:e7566. doi: 10.7717/peerj.7566 (PMC6727833; doi:10.7717/peerj.7566)
Supplement: Supplemental Information 4 [file peerj-07-7566-s004.pdf]

Appendix S4: Adjacency matrices and network structure for a network with mixed modules

Figure D1. Adjacency matrices and network structure for a network with mixed modules. Sampling occurred on *m*=50 anchor nodes and adding up to 10 first neighbors. The network has 16 modules with sizes 67, 18, 21, 20, 20, 32, 20, 20, 16, 16, 50, 58, 12, 64, 25 and 41. The average degree is 11.8 and average module size is 31.25. Anchor nodes were chosen randomly (a)-(b), according to degree (c)-(d) or to according to module (e)-(f). Nodes and links in red represent the sampled species and interactions in each case. The number of connected components is 7, 5 and 10 respectively. Note that some modules are completely missed by sampling, such as modules 1, 11 and 12 (counting from bottom left to top right in the adjacency matrix) in (e)-(f).

(a) (b)

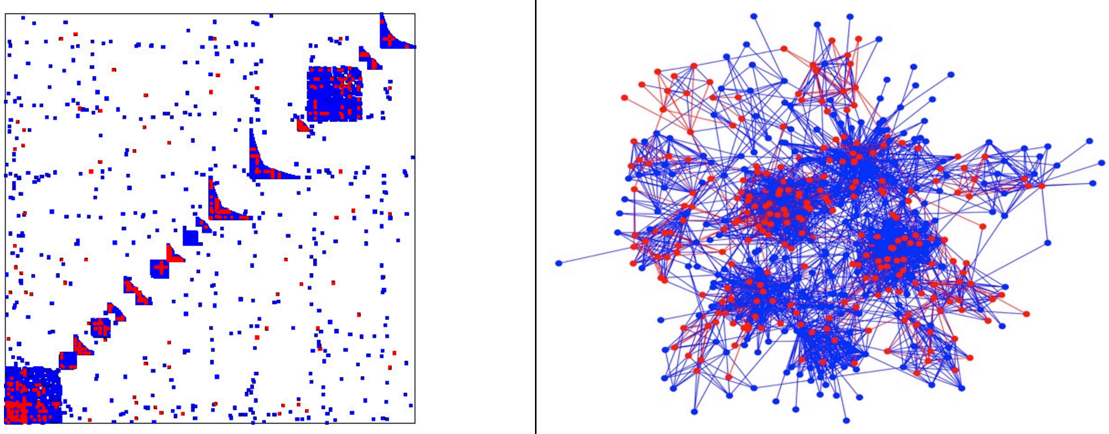

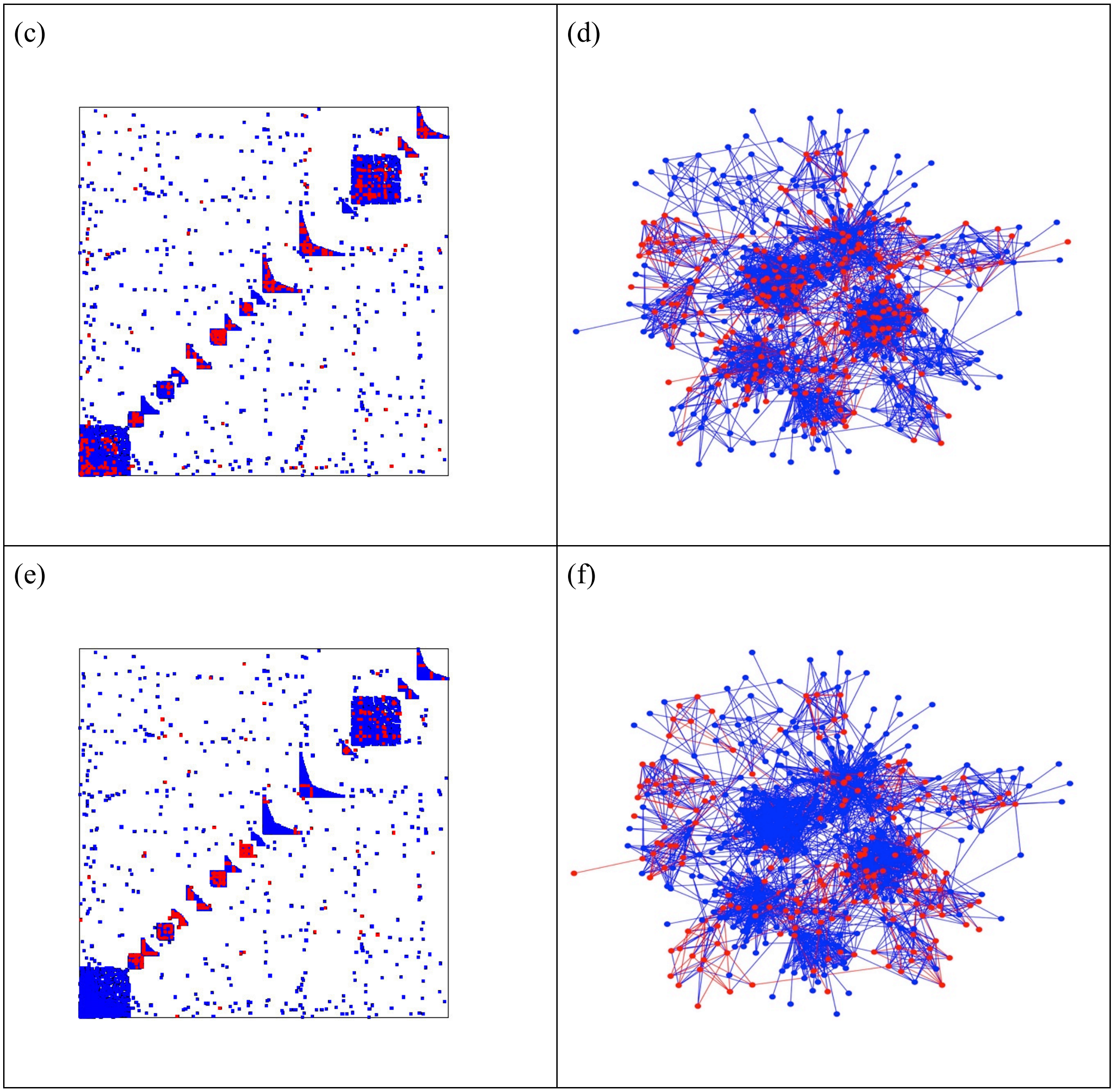

(c)
(d)
(e)
(f)
