# Supplementary material for: Revealing biases in the sampling of ecological interaction networks
Source: PeerJ. 2019 Sep 2;7:e7566. doi: 10.7717/peerj.7566 (PMC6727833; doi:10.7717/peerj.7566)
Supplement: Supplemental Information 5 [file peerj-07-7566-s005.pdf]

Appendix S5: *EcoNetGen*, including *NetGen* and *NetSampler* Python and Fortran scripts

EcoNetGen contains *NetGen* and *NetSampler*, which are software scripts that run in Python. Each script calls another script from Fortran (both languages must be installed to run either script). All code is freely available as file s5_EcoNetGen_Py.zip. Table E1 shows the output files of *NetGen* and *NetSampler*.

Table E1. Output files of Python scripts *NetGen* and *NetSampler* in *EcoNetGen*, with descriptions and locations to which the files are written with each run of the scripts

Software	Files Generated	Location	Description
NetGen 1.0	network_net.txt	output_gen	contains a list of pairs of nodes that are connected and can be used to plot the network's structure using python packages like networkx
	network_adj.txt	output_gen	contains the adjacency matrix
	network_prop.txt	output_gen	contains a list of the module sizes and types followed by a short summary of the network properties. This information is also printed on the screen
	network_net.png	output_gen	graphic visualization of the network in .png format
	network_adj.png	output_gen	graphic visualization of the adjacency matrix in .png format
	log_gen.txt	output_gen	log file containing network-name and other network properties. The file is updated automatically after each run of *NetGen*
NetSampler 1.0	network_auxname_sampled.txt	output_sampled	contains a list of pairs of nodes that are connected in the sampled network
	network_auxname_nodes.txt	output_sampled	contains a list of nodes in the original network followed by a color. Default colors are red for sampled nodes and blue for nodes that were not sampled
	network_auxname_links.txt	output_sampled	contains a list of all pairs of nodes that are in the original network followed by a color. Default colors are red for sampled connections and blue for connections that were not sampled
	network_auxname_full_and_sampled_net.png	output_sampled	graphic visualization of the sampled network (red) within the original network (blue) in .png format

network_auxname_sampled_adj.png	output_sampled	graphic visualization of the sampled adjacency matrix (red) within the original adjacency matrix (blue) in .png format
network_auxname_sampled_net.png	output_sampled	graphic visualization of the sampled network in png format - nodes are numbered according to sampled order, with anchor nodes first
network_auxname_full_and_sampled_adj.png	output_sampled	graphic visualization of the sampled adjacency matrix in .png format. Nodes are numbered according to sampled order, with anchor nodes first
abund.txt	output_sampled	abundances attributed to each node (species) when sampling by abundances
log_sampled.txt	output_sampled	log file containing the network name, the auxname and parameters of the sampling procedure. The file is updated automatically after each run of *NetSampler*
