# Supplementary figures and images for: Revealing biases in the sampling of ecological interaction networks
Source: PeerJ. 2019 Sep 2;7:e7566. doi: 10.7717/peerj.7566 (PMC6727833; doi:10.7717/peerj.7566)

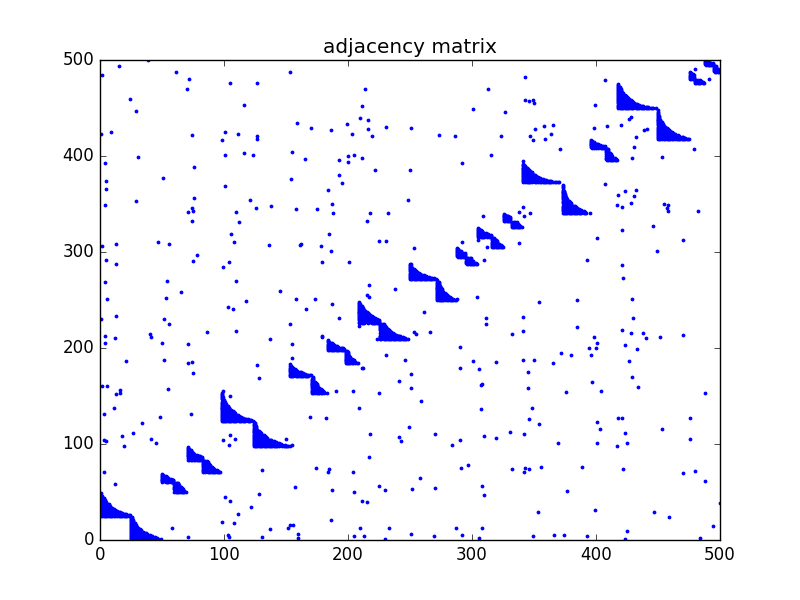

Supplement: Supplemental Information 6 [file peerj-07-7566-s006.zip › Python-Package/output_gen/bipartite_adj.png]

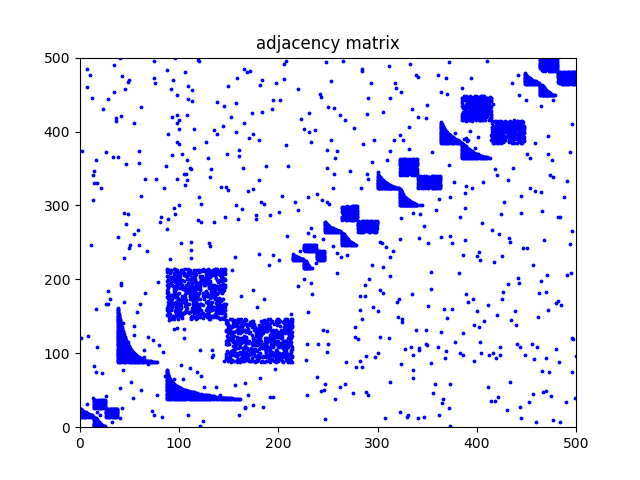

Supplement: Supplemental Information 6 [file peerj-07-7566-s006.zip › Python-Package/output_gen/tripartite_adj.png]

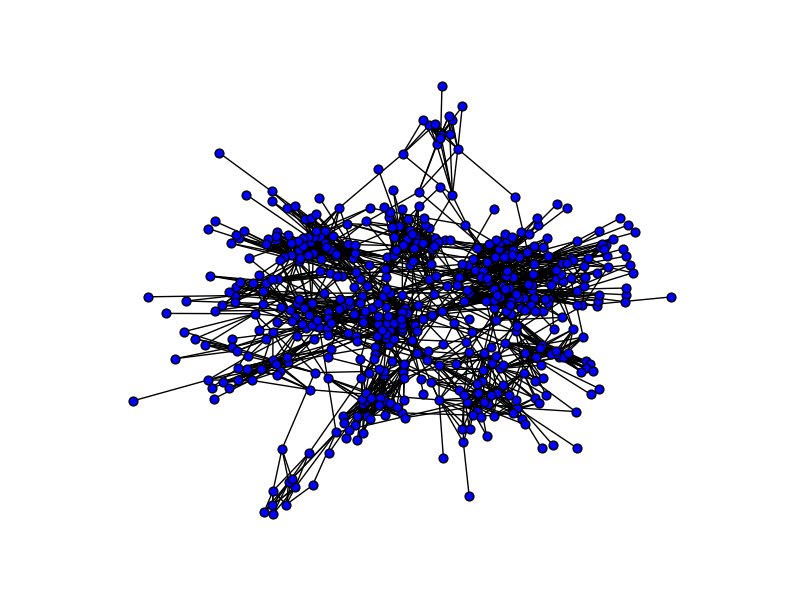

Supplement: Supplemental Information 6 [file peerj-07-7566-s006.zip › Python-Package/output_gen/bipartite_net.png]

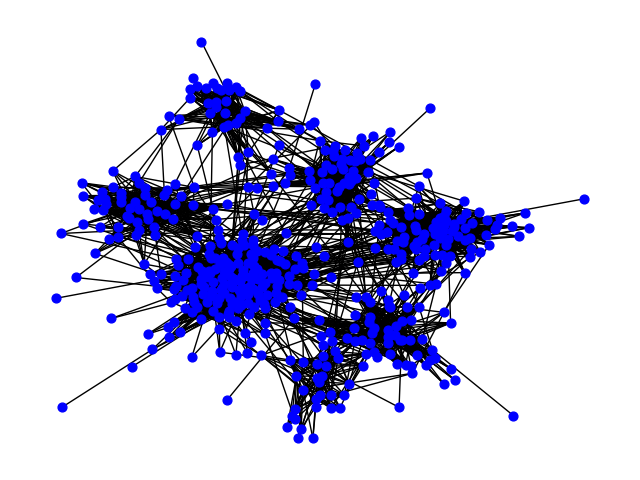

Supplement: Supplemental Information 6 [file peerj-07-7566-s006.zip › Python-Package/output_gen/tripartite_net.png]

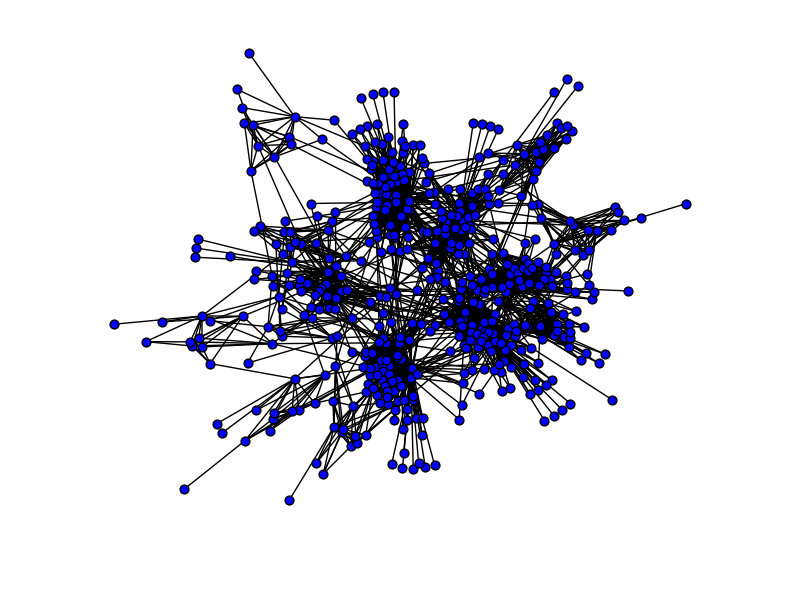

Supplement: Supplemental Information 6 [file peerj-07-7566-s006.zip › Python-Package/output_gen/nested_net.png]

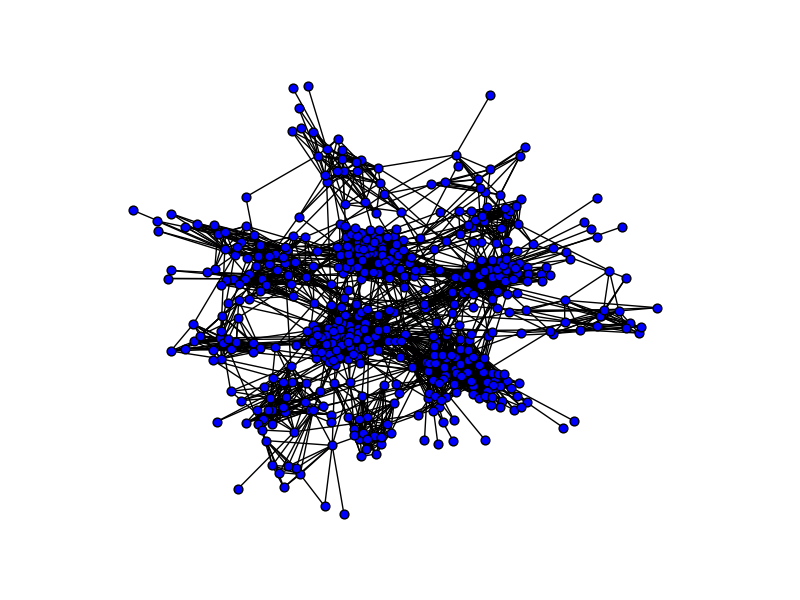

Supplement: Supplemental Information 6 [file peerj-07-7566-s006.zip › Python-Package/output_gen/mixed_net.png]

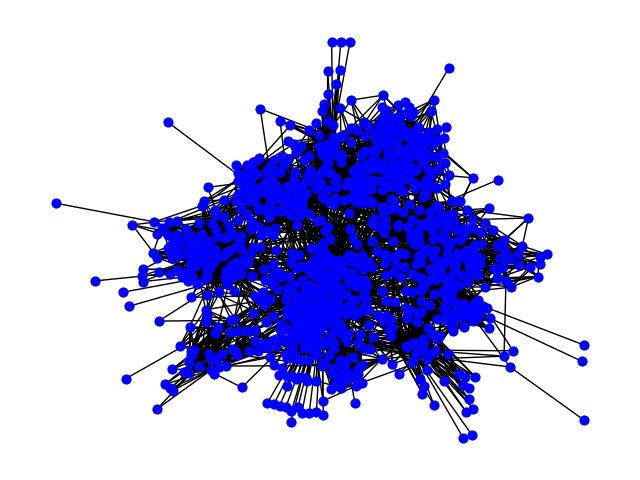

Supplement: Supplemental Information 6 [file peerj-07-7566-s006.zip › Python-Package/output_gen/mixed3_net.png]

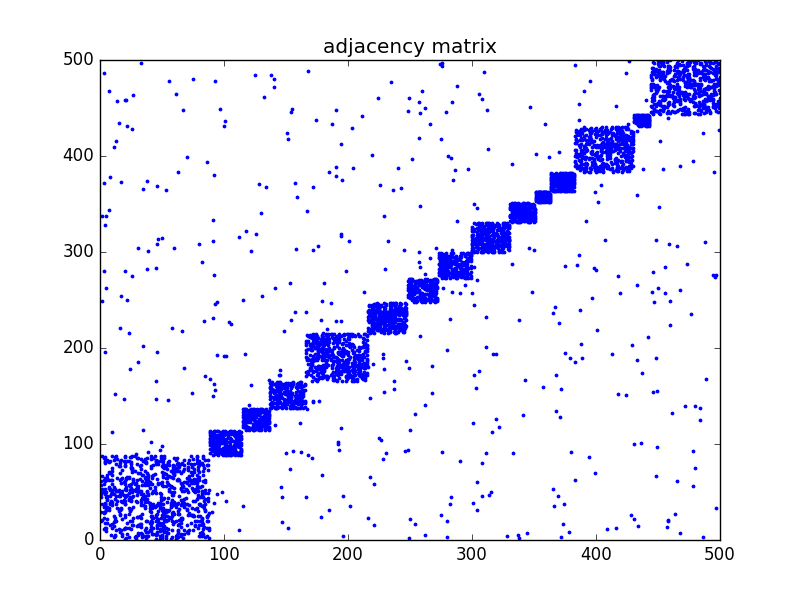

Supplement: Supplemental Information 6 [file peerj-07-7566-s006.zip › Python-Package/output_gen/random_adj.png]

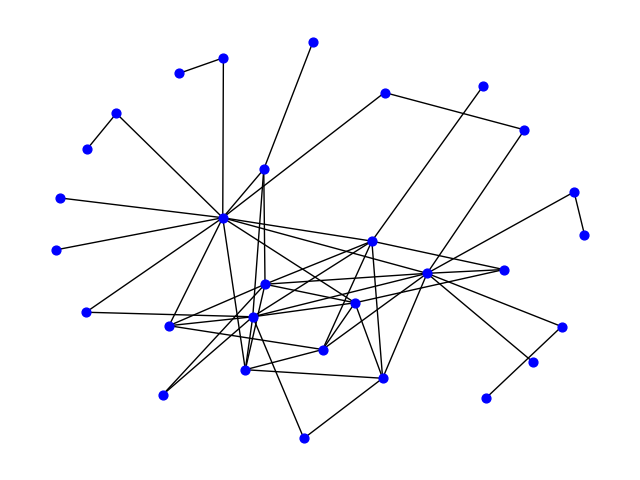

Supplement: Supplemental Information 6 [file peerj-07-7566-s006.zip › Python-Package/output_gen/bn30_net.png]

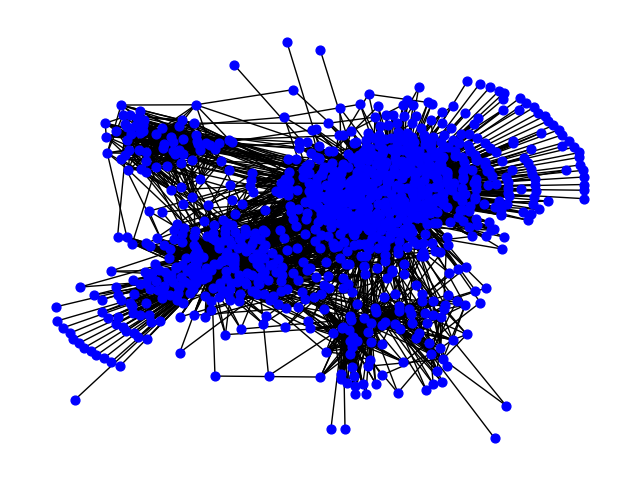

Supplement: Supplemental Information 6 [file peerj-07-7566-s006.zip › Python-Package/output_gen/mixed2_net.png]

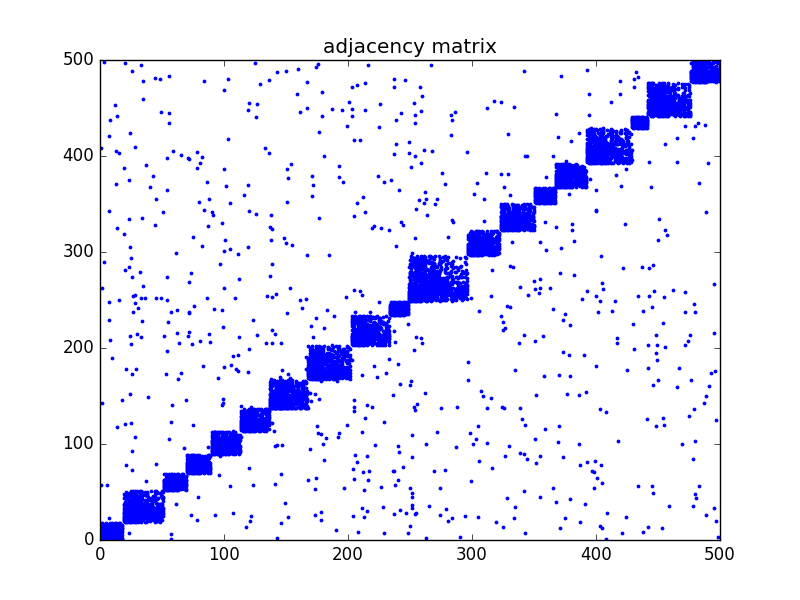

Supplement: Supplemental Information 6 [file peerj-07-7566-s006.zip › Python-Package/output_gen/scalefree_adj.png]

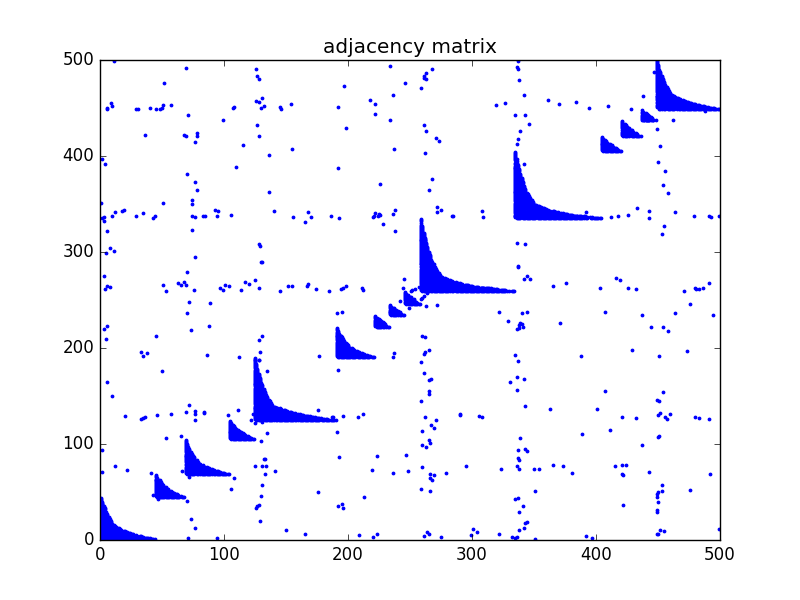

Supplement: Supplemental Information 6 [file peerj-07-7566-s006.zip › Python-Package/output_gen/nested_adj.png]

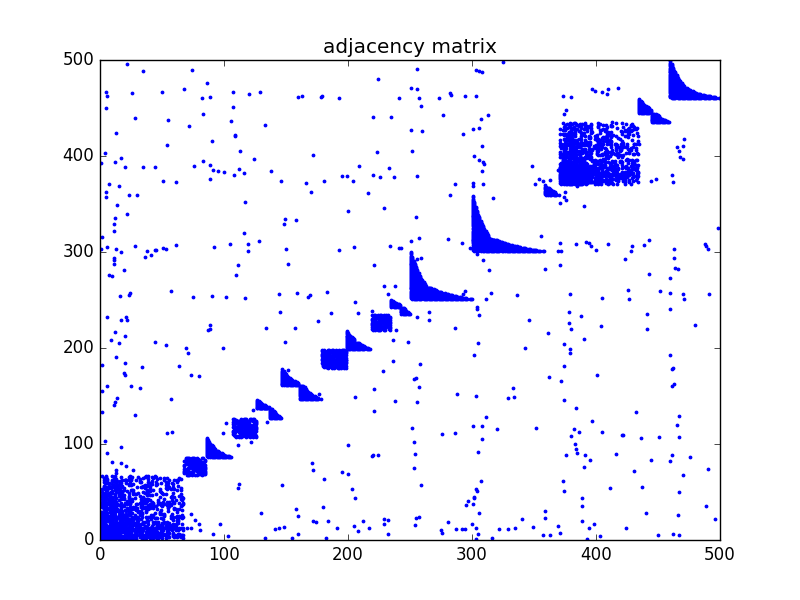

Supplement: Supplemental Information 6 [file peerj-07-7566-s006.zip › Python-Package/output_gen/mixed_adj.png]

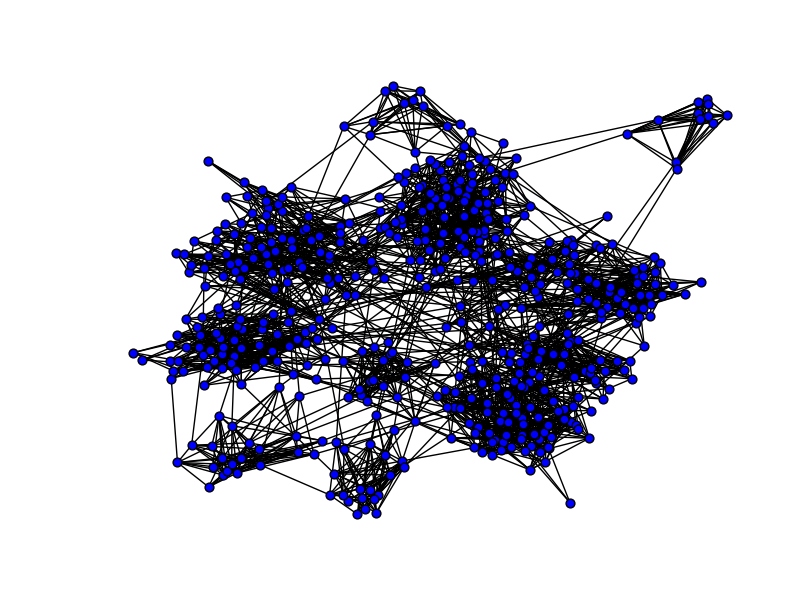

Supplement: Supplemental Information 6 [file peerj-07-7566-s006.zip › Python-Package/output_gen/random_net.png]

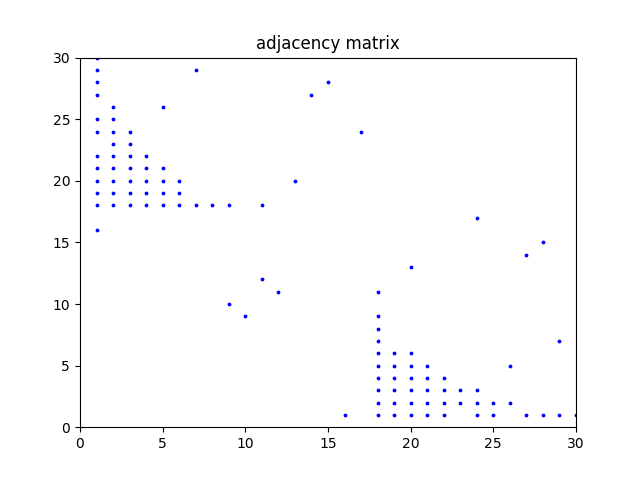

Supplement: Supplemental Information 6 [file peerj-07-7566-s006.zip › Python-Package/output_gen/bn30_adj.png]

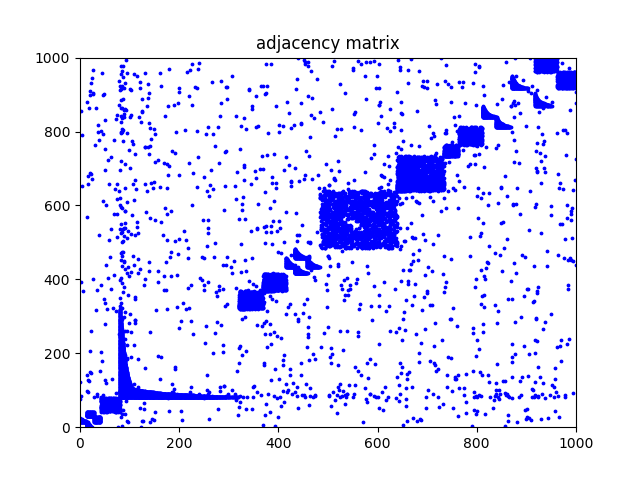

Supplement: Supplemental Information 6 [file peerj-07-7566-s006.zip › Python-Package/output_gen/mixed3_adj.png]

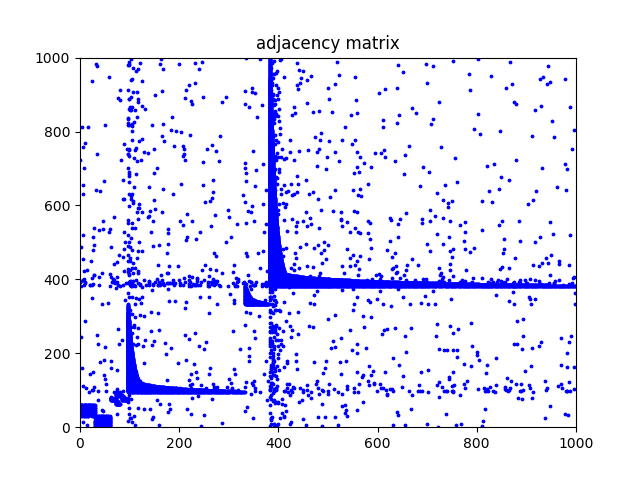

Supplement: Supplemental Information 6 [file peerj-07-7566-s006.zip › Python-Package/output_gen/mixed2_adj.png]

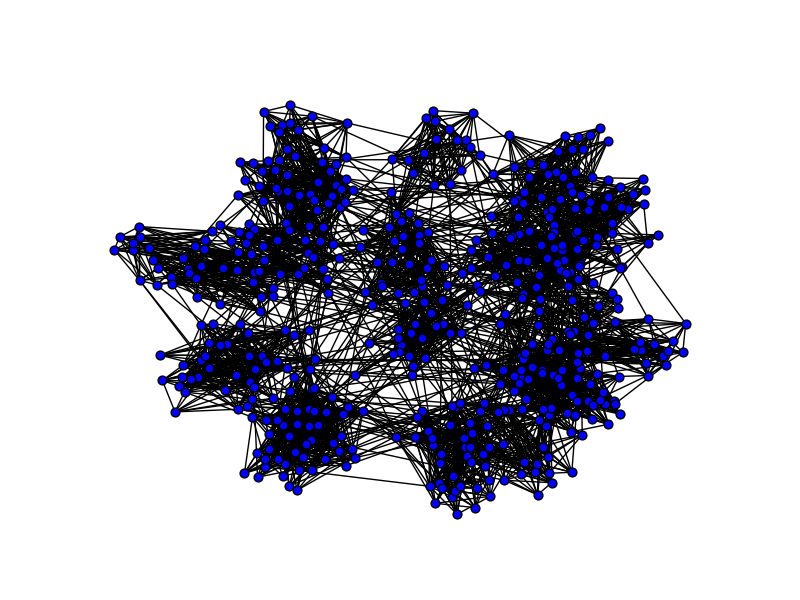

Supplement: Supplemental Information 6 [file peerj-07-7566-s006.zip › Python-Package/output_gen/scalefree_net.png]

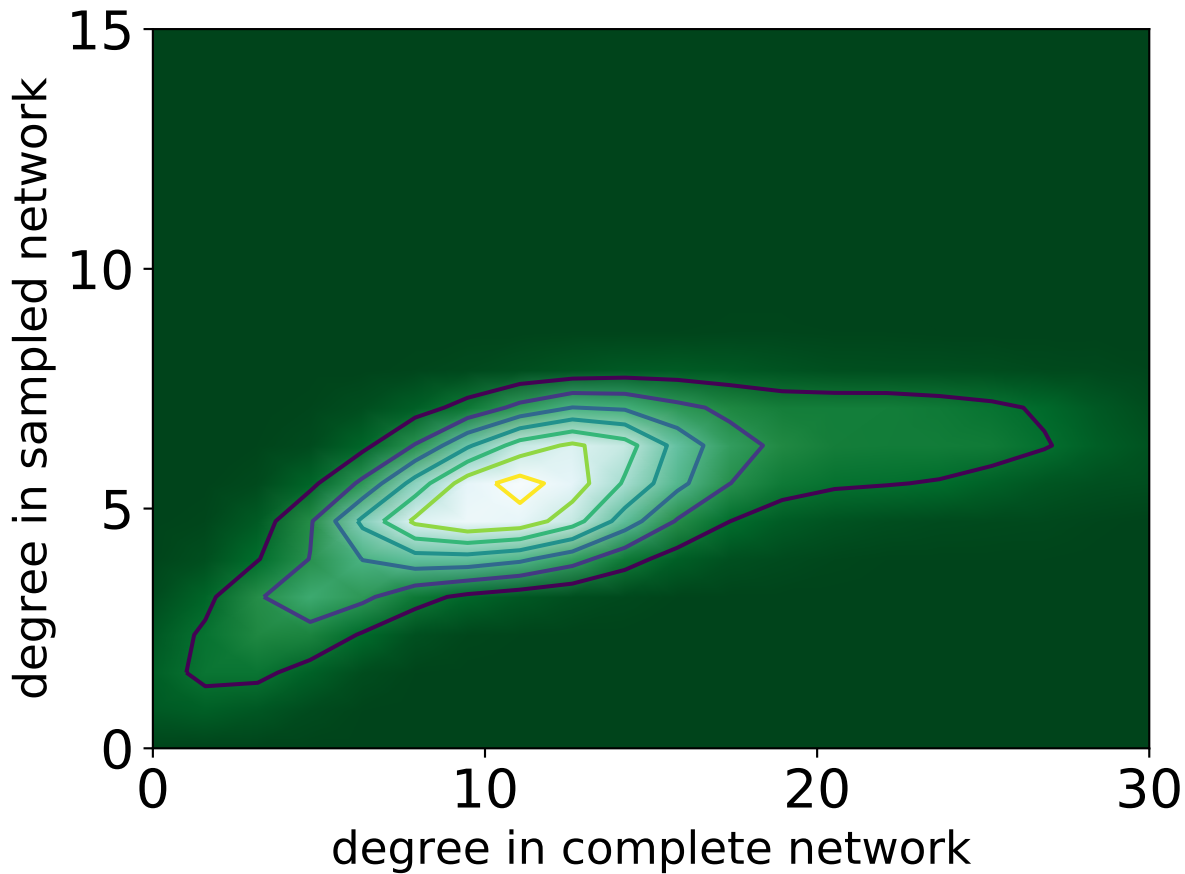

Supplement: Supplemental Information 6 [file peerj-07-7566-s006.zip › Python-Package/output_sampled/tripartite-modnfn7-module.pdf]

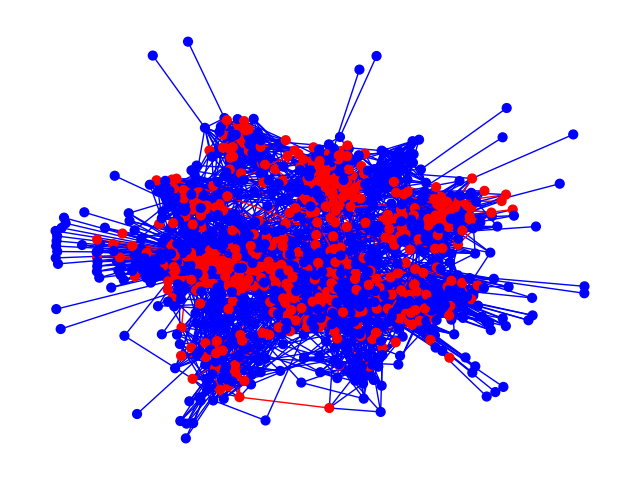

Supplement: Supplemental Information 6 [file peerj-07-7566-s006.zip › Python-Package/output_sampled/mixed3_modnfn10-degree_full_and_sampled_net.png]

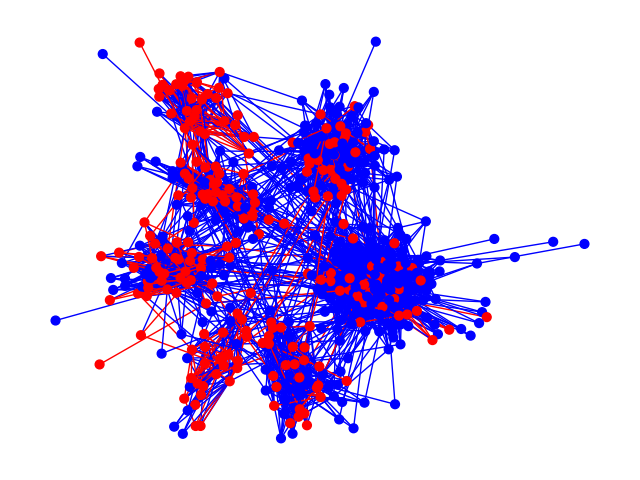

Supplement: Supplemental Information 6 [file peerj-07-7566-s006.zip › Python-Package/output_sampled/tripartite_modnfn7-module_full_and_sampled_net.png]

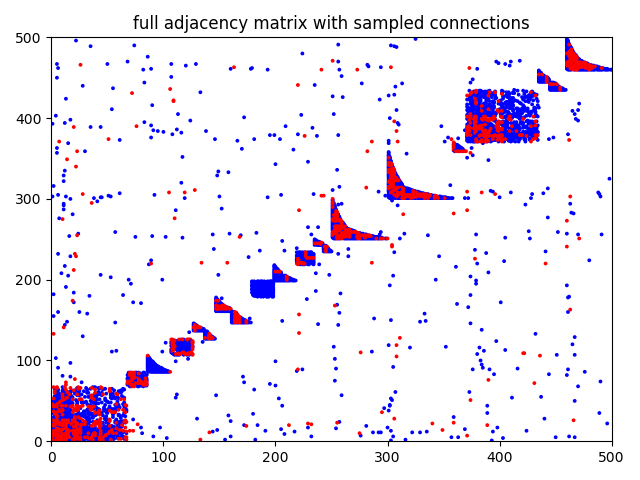

Supplement: Supplemental Information 6 [file peerj-07-7566-s006.zip › Python-Package/output_sampled/mixed_modnfn10-degree_full_and_sampled_adj.png]

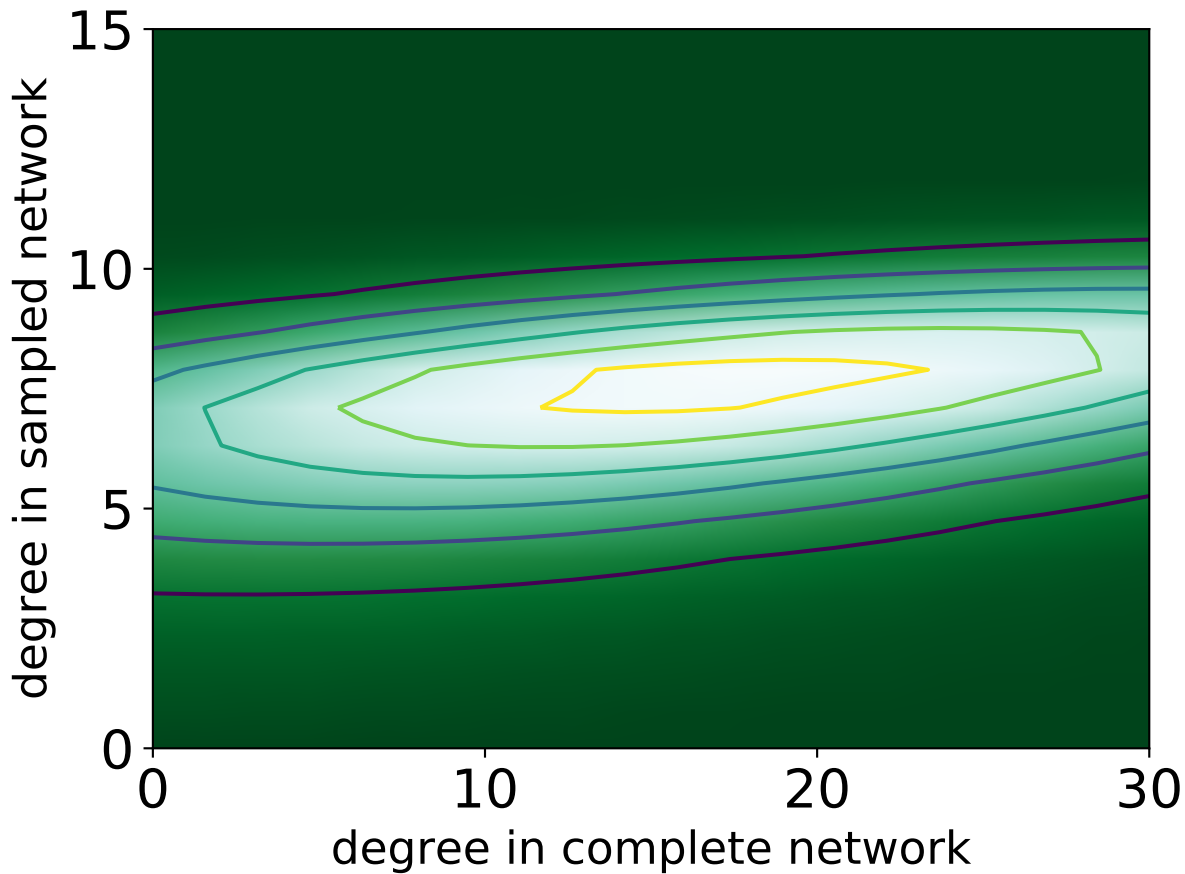

Supplement: Supplemental Information 6 [file peerj-07-7566-s006.zip › Python-Package/output_sampled/mixed3-modnfn10-degree.pdf]

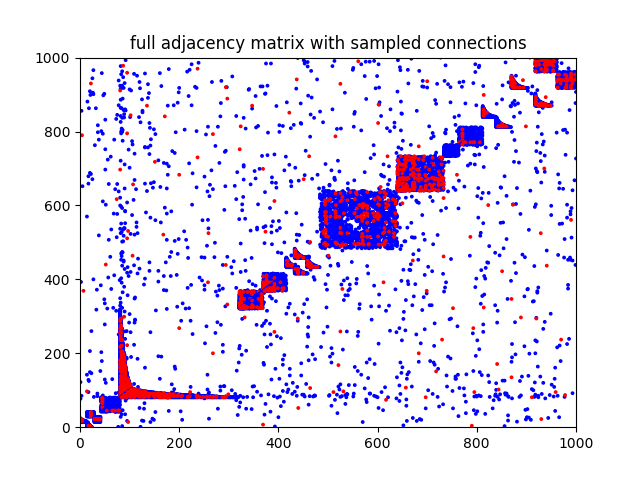

Supplement: Supplemental Information 6 [file peerj-07-7566-s006.zip › Python-Package/output_sampled/mixed3_modnfn10-degree_full_and_sampled_adj.png]

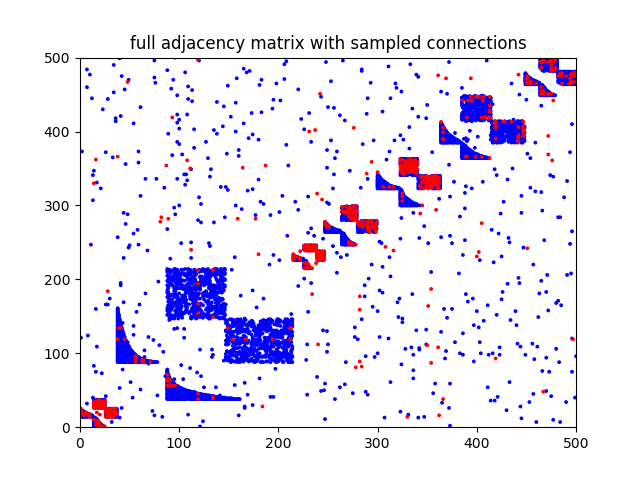

Supplement: Supplemental Information 6 [file peerj-07-7566-s006.zip › Python-Package/output_sampled/tripartite_modnfn7-module_full_and_sampled_adj.png]

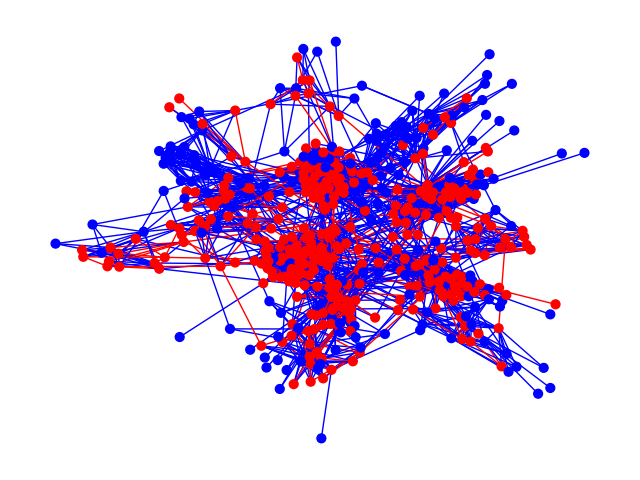

Supplement: Supplemental Information 6 [file peerj-07-7566-s006.zip › Python-Package/output_sampled/mixed_modnfn10-degree_full_and_sampled_net.png]

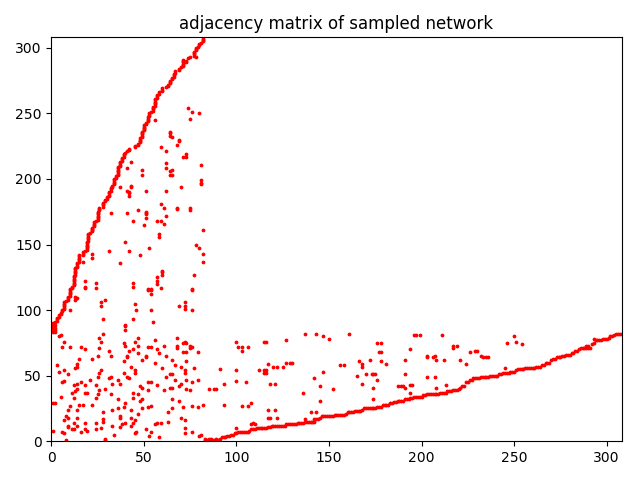

Supplement: Supplemental Information 6 [file peerj-07-7566-s006.zip › Python-Package/output_sampled/bipartite_m50nfn10-degree_sampled_adj.png]

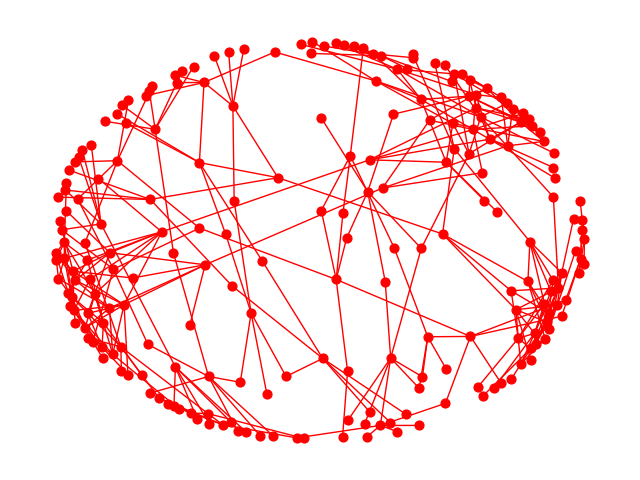

Supplement: Supplemental Information 6 [file peerj-07-7566-s006.zip › Python-Package/output_sampled/tripartite_modnfn7-module_sampled_net.png]

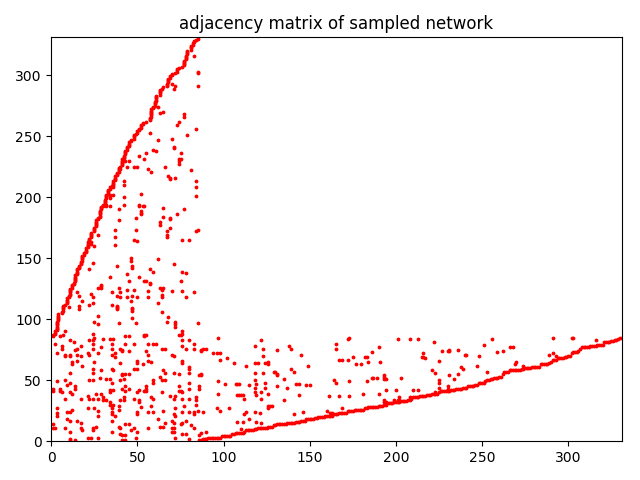

Supplement: Supplemental Information 6 [file peerj-07-7566-s006.zip › Python-Package/output_sampled/mixed_modnfn10-degree_sampled_adj.png]

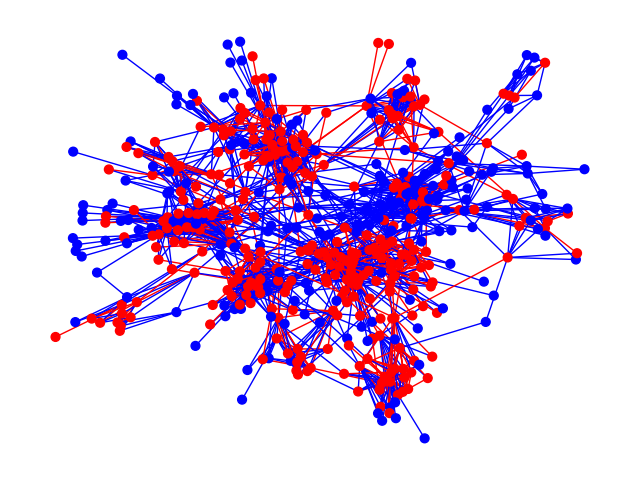

Supplement: Supplemental Information 6 [file peerj-07-7566-s006.zip › Python-Package/output_sampled/bipartite_m50nfn10-degree_full_and_sampled_net.png]

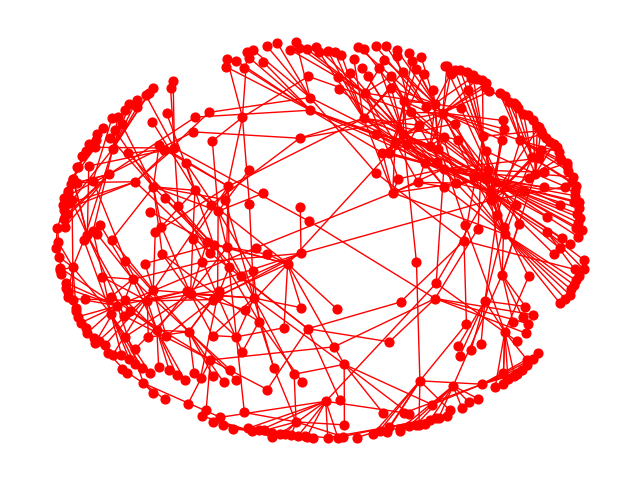

Supplement: Supplemental Information 6 [file peerj-07-7566-s006.zip › Python-Package/output_sampled/mixed3_modnfn10-degree_sampled_net.png]

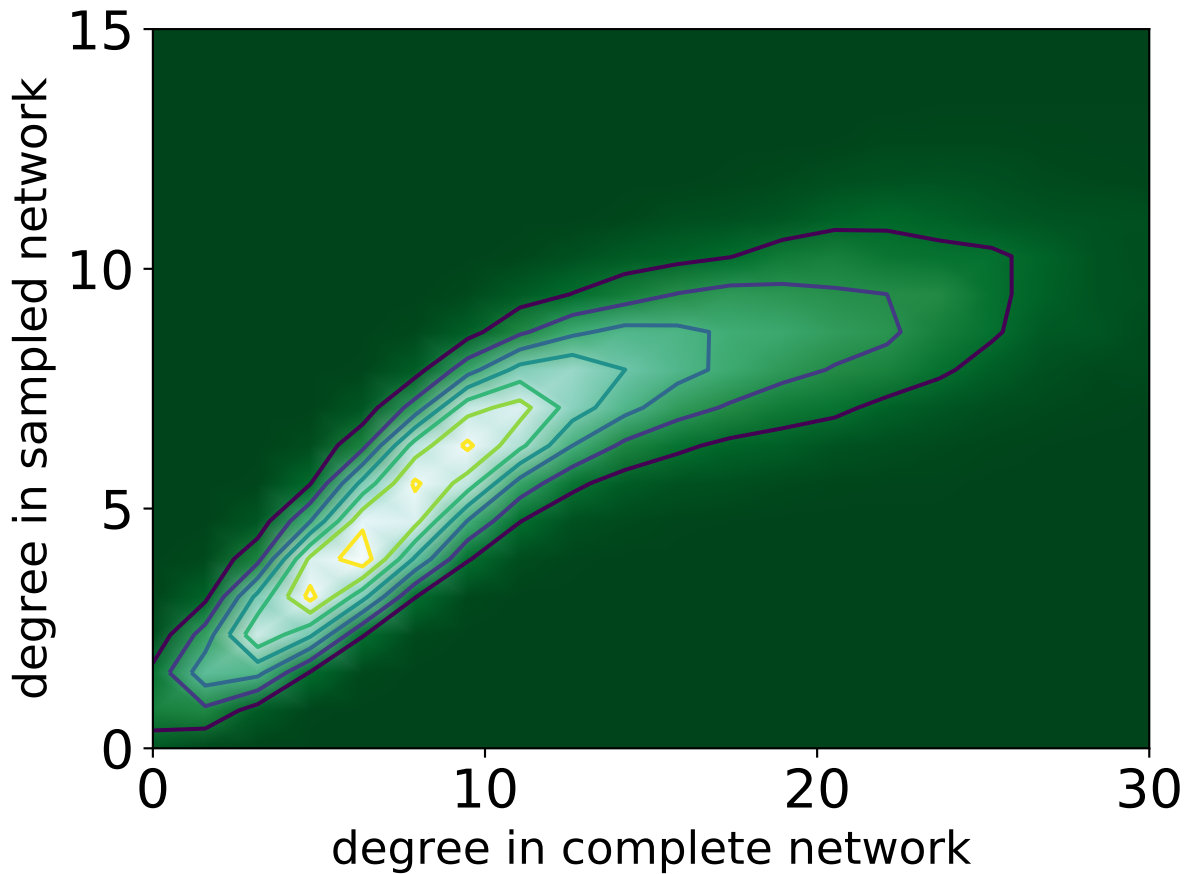

Supplement: Supplemental Information 6 [file peerj-07-7566-s006.zip › Python-Package/output_sampled/bipartite-m50nfn10-degree.pdf]

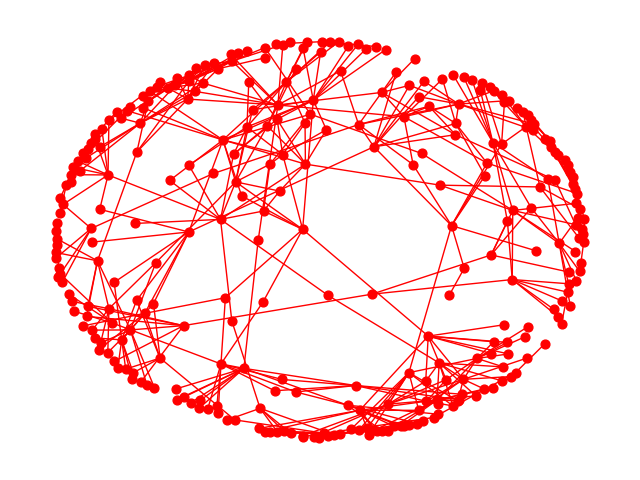

Supplement: Supplemental Information 6 [file peerj-07-7566-s006.zip › Python-Package/output_sampled/bipartite_m50nfn10-degree_sampled_net.png]

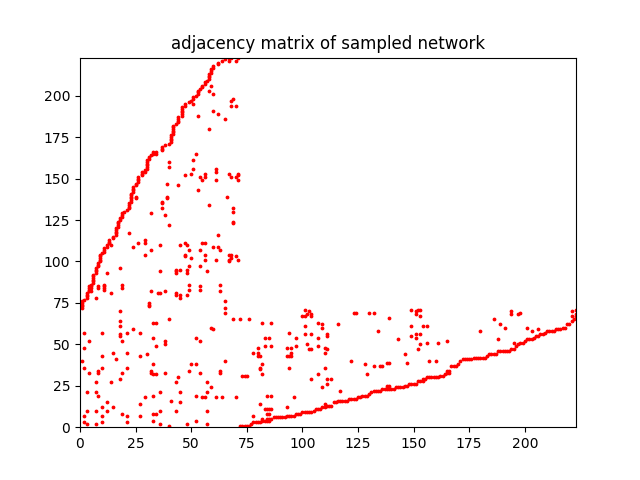

Supplement: Supplemental Information 6 [file peerj-07-7566-s006.zip › Python-Package/output_sampled/tripartite_modnfn7-module_sampled_adj.png]

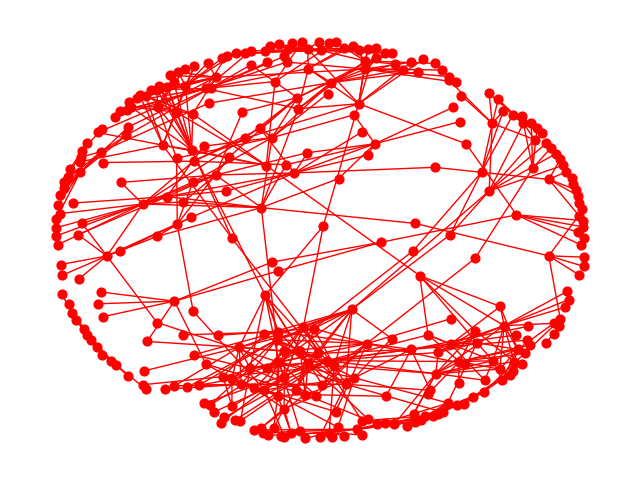

Supplement: Supplemental Information 6 [file peerj-07-7566-s006.zip › Python-Package/output_sampled/mixed_modnfn10-degree_sampled_net.png]

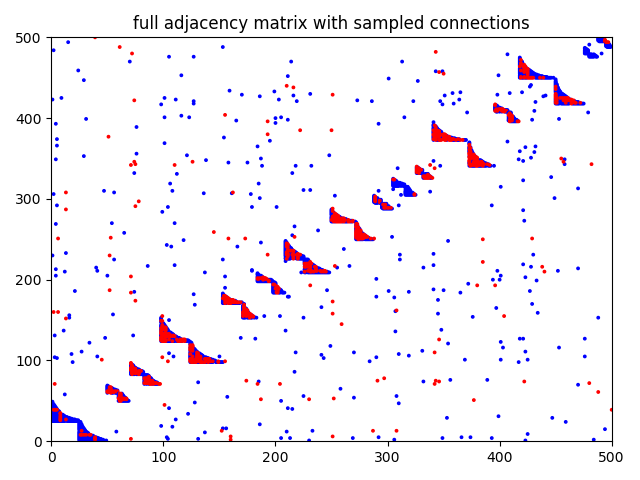

Supplement: Supplemental Information 6 [file peerj-07-7566-s006.zip › Python-Package/output_sampled/bipartite_m50nfn10-degree_full_and_sampled_adj.png]

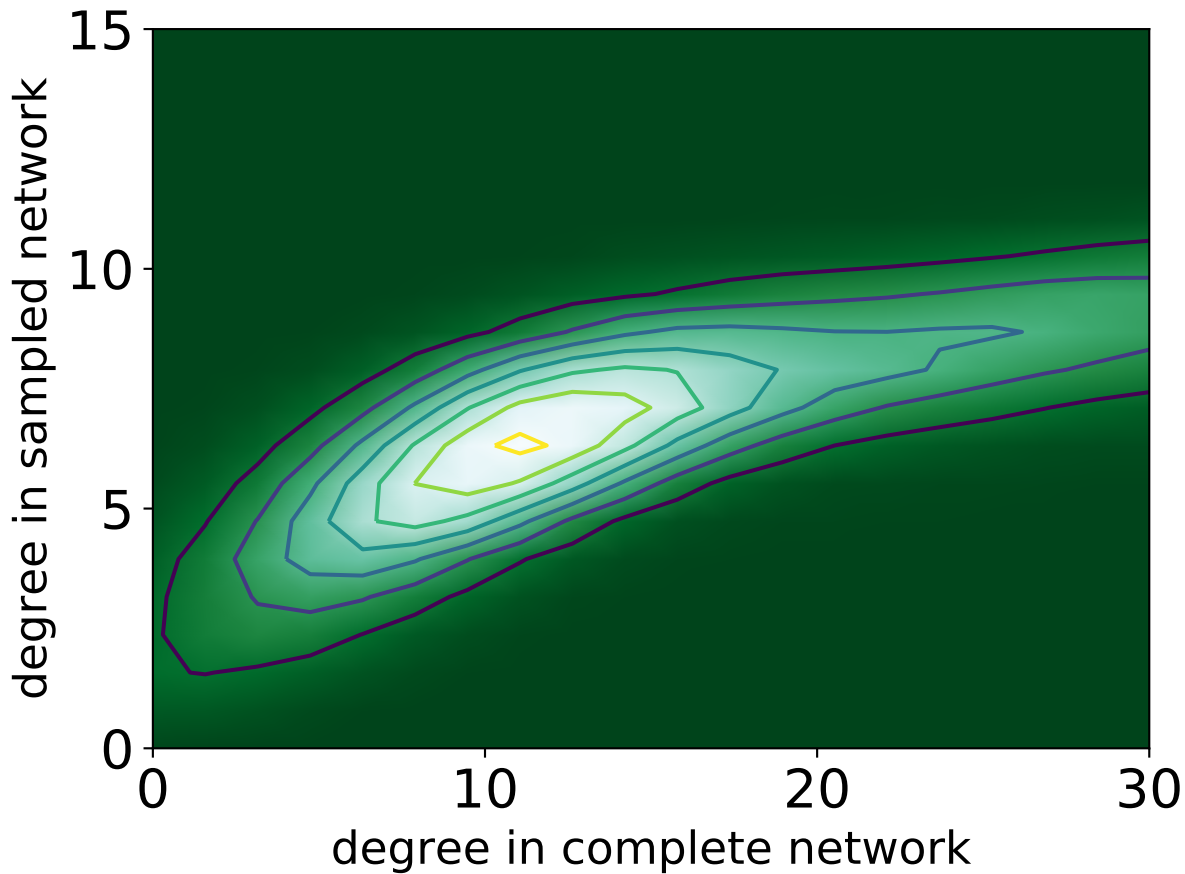

Supplement: Supplemental Information 6 [file peerj-07-7566-s006.zip › Python-Package/output_sampled/mixed-modnfn10-degree.pdf]

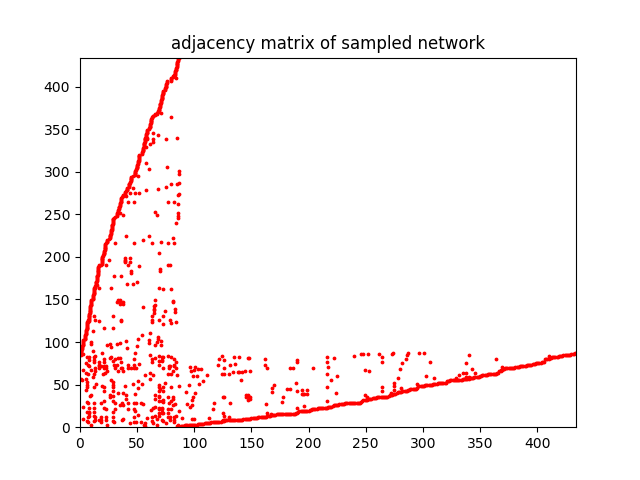

Supplement: Supplemental Information 6 [file peerj-07-7566-s006.zip › Python-Package/output_sampled/mixed3_modnfn10-degree_sampled_adj.png]
